# Supplementary material for: Puerto Ricans and Mexican immigrants differ in their psychological responses to patterns of lifetime adversity
Source: PLoS One. 2021 Oct 18;16(10):e0258324. doi: 10.1371/journal.pone.0258324 (PMC8523059; doi:10.1371/journal.pone.0258324)
Supplement: Attachment — Submitted filename: Latinxs_Adversity_MH_PLOSONE.pdf [file pone.0258324.s001.pdf]

Overall Comments:

This study examines dimensionality of adverse childhood experiences and perceived discrimination on the mental health risk among Mexican and Puerto Rican adults living in the US. The study offers a unique contribution in this area of research as the study, (1) assesses ACEs among rarely assessed Latinx-samples, specifically, (2) teases apart unique patterns of adversities versus past research that bundles all adversity together, (3) includes a rich dataset from the broader SOL study, and (4) conducts mental health research among Latinx populations that disaggregates versus typical studies that examine the group homogenously. The manuscript could benefit from much greater attention to the necessary theoretical background to this very important topic, as well as providing more clear, precise writing about the populations, patterns, and processes, etc. that are relevant to this study. Also, limitations of this study's findings to the specific groups that are included in this study, and therefore the specific childhood contexts should be described, as these childhood contexts by and large exclude ACE-related experience occurring in the continental US for continental US-born/raised Latinx populations. I hope my comments below are useful to the authors in order to strengthen their manuscript further.

Abstract:

1. As an example of adversity across the lifespan, "childhood adversity" reads as redundant and should be replaced with specific relevant adversities instead such as violence or poverty during childhood, interpersonal/structural discrimination across the lifespan, etc.
2. It should be made clear whether mental health problems being assessed are lifetime, current, or adulthood only.
3. For readers unfamiliar or with alternative views regarding labels, it is unclear the motivation for using Latinx in the Background when the study is restricted to mainland Puerto Rican and Mexican Latinx adults, specifically. Further, it is incorrect to refer to Puerto Rican and Mexican Latinx adults living in the continental US as 'immigrants' because (1) Puerto Rican adults living in the continental US are all US citizens at birth regardless whether they are born in the island or mainland US, and (2) Mexican adults living in the continental US may include a combination of US-born and immigrant populations. In general, the population of focus is unclear in the Abstract.

Introduction:

1. 1st paragraph, Page 3: It is unclear how prevalence of ACEs among Latinxs compare to other groups to assess whether 77% is more or less than average. It is also unclear what Latinx groups that this 77% includes – gender/sexual minorities, Mexican/Puerto Rican only as in the study, immigrant Latinx (non-US born), US-born only, etc. Do these adversities occur among US-born? Does perceived discrimination refer to interpersonal or structural as this construct has evolved considerably to tease apart different forms of discrimination and their impacts on health. Clearer definitions and statements should be provided according to the specific groups, terminology, and literature referenced.
2. 2nd paragraph, Page 3: It seems the LCT is insufficient to be the sole theory guiding this study. LCT guides the expected impacts of childhood exposures on outcomes in adulthood, including identification of sensitive periods, but doesn't capture life course

experiences/adversity that are specific to Latinx populations, that say critical race theory or weathering hypotheses could. Research by Maggie Alegria, William Vega, Hector Bird, etc. that have examined mental health epidemiology among Latinx populations specifically across the life course may support this background.

3. 3rd paragraph, Page 4-5: What is missing from childhood adversity is the validity of these measures to non-White groups thereby the focus of past research among non-white samples only. To what extent are Latinx sample included in ACE measurement studies? Also, there is more recent theory about dimensionality of ACE's effects on the brain, versus cumulative effects, that would be important to include in the background of this current study. See work by Katie A. McClaughlin et al., particularly PMID: 27773969. Finally, concerning adult adversity, literature about epigenetic effects could be included here as well as literature examining trauma which is much more developed and has demonstrated similar interactive effects between childhood ACE's and traumas/adversities during adulthood and their effects on mental health (e.g., disaster-related and veterans-related research examining ACE's plus adulthood adversities and their impacts on risk for post-traumatic stress).
4. 5th paragraph, page 5-6: The background on discrimination would benefit from describing the different kinds of discrimination (interpersonal/individual versus structural) and their impacts on health. Then it should be explicitly clear that this study examines individual only. It is also noted variation in experiences based on race/skin color within Latinx groups. To readers unfamiliar, it may be important to note the diversity within the Latinx group by race, ethnicity, language use, immigration status, geography, etc. that inform discrimination experiences. Also wouldn't we expect intersectionality to matter here for Latinx who are black, immigrant, and/or gender/sexual minority such that interacting, multi-layered, co-occurring discrimination experiences present unique threats to health versus discrimination among white-passing, cis-gendered, US-born Latinx populations? Greater acknowledgement of these intersections can help move the knowledge base forward in this area (see original work by Kimberle Crenshaw for more information).
5. Concerning Latinx heterogeneity, this text should be introduced much earlier to help guide the reader as to why we would expect variation in the application of ACEs and LCT in research pertaining to Latinx populations. Also, it is unclear why Mexican and Puerto Rican groups are of focus – is only because they are the largest sub-groups or is it that other groups are underrepresented in research? How does research on Mexican and Puerto Rican populations generalize to other groups? What are the public mental health impacts of focusing on the two largest sub-groups only? How do these two groups differ in terms of other socio-demographics such as race, age, immigration status, language use, etc. (PEW Hispanic Research Center may provide this information). Why determine highest risk groups for tailored intervention versus low-dose, ubiquitous interventions for all (e.g., universal mental health coverage, Spanish-language competent providers, etc.) – what are the tradeoffs of one approach versus another? These motivations should be made clearer.

Methods:

1. Sample, Page 9: It is unclear the motivation for restricting the sample to two Latinx groups only. It is also unclear from the Abstract and Introduction if this study and Background is

pertaining to Puerto Rican and Mexican adults living in the mainland US who were not born in the mainland US, specifically.

2. The only reason provided, "These were two of the largest Latinx subgroups in the sample and represent the two largest Latinx subgroups living in the U.S." is still unclear in terms of what is meant by "subgroup". Ethnic subgroup? Among immigrant populations? Among non-mainland US born populations? These definitions and rationale for inclusion criterion are unclear yet they should be explicit throughout the manuscript. Also, if 1753 of the 4645 were included only, how did the excluded sample compare to those that were included? Why were they excluded and why not assess South American and Central American and Caribbean groups separately?
3. Page 9-10: What is the validity of the ACE, discrimination, and mental health measures among the included Latinx populations in this study, specifically? That is, Puerto Rican and Mexican adults living in the mainland US who were not born in the mainland US. Also, alpha reliability of each measure would be helpful to include in the text.
4. Are there covariates available to assess racial/skin color variation as this would be highly relevant to the discrimination measures? Also, potential buffers or resilience factors such as health insurance status, education, and close relationships that mitigate risks of adversity/discrimination on mental health should be included. Further, is state/geography examined as CA/NY present more immigrant-favorable policies versus FL, etc. (e.g., Hatzenbuehler et al. study cited regarding state immigration policies and mental health).

Discussion:

1. Page 25-26: US citizenship status comes with greater access to health insurance as well, therefore, access to mental health care, which can mitigate negative effects of discrimination on mental health among Puerto Ricans. In contrast, those without access or without a public insurance option may be more prevalent among Mexican immigrants, particularly those that are undocumented. Nevertheless, racial/skin color variation isn't taken into account here when describing differences between Puerto Rican and Mexican discrimination experiences.
2. Limitations of the sample should be elaborated as this is a highly specific sample. There are many unique Latinx groups excluded from this study including, but not limited to, US-born Latinx, Gen Z and Millennial Latinx, gender/sexual minorities (gender assessed as binary variable), no racial variation captured, rural/suburban Latinx populations (different from new settlement areas as indicated), indigenous Latinx, as well as Central/South American and Other Caribbean groups. That US-born, young Latinx are excluded limits generalizability of these findings to understanding ACE's that occur in the continental US context, specifically. The ACE's captured in this study reflect those that occur in Mexico or the island of Puerto Rico, and *not* childhood adversities that are experienced in the continental US for other Latinx populations. Further, time spent back and forth between Mexico or Puerto Rico and mainland US during childhood, especially for Puerto Ricans who are citizens and arguably have easier migration abilities, but also Mexican border populations, isn't factored into this study. These experiences could introduce a unique set of migration-related ACE's that are not captured by the ACE measure. These important limitations should be noted.

These exclusions to the sample should also be noted, and as such, use of Latinx should be re-considered or defined.
