# Supplementary material for: Puerto Ricans and Mexican immigrants differ in their psychological responses to patterns of lifetime adversity
Source: PLoS One. 2021 Oct 18;16(10):e0258324. doi: 10.1371/journal.pone.0258324 (PMC8523059; doi:10.1371/journal.pone.0258324)
Supplement: Attachment — Submitted filename: Response to Reviewers.docx [file pone.0258324.s002.docx]

**Responses to Reviewers**

**Editor’s Comments**

1. Please ensure that your manuscript meets PLOS ONE's style requirements, including those for file naming. The PLOS ONE style templates can be found at https://journals.plos.org/plosone/s/file?id=wjVg/PLOSOne_formatting_sample_main_body.pdf and https://journals.plos.org/plosone/s/file?id=ba62/PLOSOne_formatting_sample_ title_authors_affiliations.pdf

**RESPONSE: Thank you for these useful resources. We have revised the manuscript to meet the PLOS ONE style requirements.**

2. In your revised cover letter, please address the following prompts: (a) If there are ethical or legal restrictions on sharing a de-identified data set, please explain them in detail (e.g., data contain potentially sensitive information, data are owned by a third-party organization, etc.) and who has imposed them (e.g., an ethics committee). Please also provide contact information for a data access committee, ethics committee, or other institutional body to which data requests may be sent. (b) If there are no restrictions, please upload the minimal anonymized data set necessary to replicate your study findings as either Supporting Information files or to a stable, public repository and provide us with the relevant URLs, DOIs, or accession numbers. For a list of acceptable repositories, please see <http://journals.plos.org/plosone/s/data-availability#loc-recommended-repositories>. We will update your Data Availability statement on your behalf to reflect the information you provide.

**RESPONSE: Thank you for these helpful directions. We have addressed these prompts in our cover letter.**

**Reviewer #1’s Comments**

3. Introduction: Please, be clearer about the difference between the present paper and the paper by the authors references as [33], since there may be some overlaps such as partially the same sample and depression as an outcome variable.

**RESPONSE: Thank you, great suggestion. We have added content to clarify the ways in which our study builds on our previous paper on p. 8, “*The present study aimed to build on Authors’ [36] study using a person-centered approach to determine whether unique combinations of lifetime adversity were associated with distinct mental health outcomes for Latinxs born outside the continental U.S. Specifically, our goal was to conduct a detailed comparison of two Latinx groups to assess whether differences in exposure patterns to lifetime adversity influenced individuals’ responses to adversities within these two groups*.”**

4. Methods: Please, also present average scores for the PEDQ, since that would facilitate the appraisal of the scores. It was unclear whether only immigrants were included or also persons that were born in the U.S. Please provide more information regarding this issue.

**RESPONSE: Based on further consideration of the literature and your comment, we chose to update the analysis using mean scores for each of the dimensions of perceived discrimination.**

**We have revised the manuscript and added content to clarify our sample on p. 8, “*The present study aimed to build on Authors’ [36] study using a person-centered approach to determine whether unique combinations of lifetime adversity were associated with distinct mental health outcomes for Latinxs born outside the continental U.S. Specifically, our goal was to conduct a detailed comparison of two Latinx groups to assess whether differences in exposure patterns to lifetime adversity influenced individuals’ responses to adversities within these two groups. Therefore, we selected Puerto Ricans and Mexicans born outside the continental U.S. because they represented some of variability that exists between U.S. Latinx groups (e.g., immigrant status, skin color, acculturation levels) and are the two largest Latinx ethnic groups living in the U.S.”***

5. Tables: I would suggest to add numbers to the first column in Table 2, in order to facilitate the attribution of the numbers found in the first line to the variables presented in the first column.

**RESPONSE: We have added numbers to the first columns of Tables 2 and 3.**

**Reviewer #2’s Comments**

1. The manuscript could benefit from much greater attention to the necessary theoretical background to this very important topic, as well as providing more clear, precise writing about the populations, patterns, and processes, etc. that are relevant to this study.

**RESPONSE: Thank you for this comment, we agree. We have revised the introduction and overall paper to improve the theoretical grounding for our study and the clarity and consistency of our terminology. We discuss the specific changes we made in our responses to comments below.**

2. Also, limitations of this study’s findings to the specific groups that are included in this study, and therefore the specific childhood contexts should be described, as these childhood contexts by and large exclude ACE-related experience occurring in the continental US for continental US-born/raised Latinx populations. I hope my comments below are useful to the authors in order to strengthen their manuscript further.

**RESPONSE: Thank you. We have clarified the populations to which our study is generalizable in the limitations section. Please see our responses to comments 23-25. Please let us know if you would like us to include any other information.**

3. Abstract: As an example of adversity across the lifespan, “childhood adversity” reads as redundant and should be replaced with specific relevant adversities instead such as violence or poverty during childhood, interpersonal/structural discrimination across the lifespan, etc.

**RESPONSE: Thank you for this suggestion. We have revised the sentence as follows, “*Puerto Ricans and Mexican immigrants are often exposed to multiple types of adversity across their lifetime (e.g., maltreatment, household dysfunction, discrimination) and this exposure can increase the risk for adult mental health problems.*”**

4. It should be made clear whether mental health problems being assessed are lifetime, current, or adulthood only.

**RESPONSE: Thank you. We have revised the paper to have greater clarity around the timing of mental health problems. For example, we updated the first sentence of the abstract to focus on adult mental health problems. We also revised our aims statement on p. 9, “The aims of our study were to (a) identify latent profiles of Puerto Rican and Mexican individuals exposed to unique combinations of lifetime adversity (i.e., childhood adversity and perceived discrimination) and (b) compare the prevalence and level of adult mental health problems (i.e., trait anxiety, depressive symptoms, and trait anger) based on latent profile membership.”**

5. For readers unfamiliar or with alternative views regarding labels, it is unclear the motivation for using Latinx in the Background when the study is restricted to mainland Puerto Rican and Mexican Latinx adults, specifically. Further, it is incorrect to refer to Puerto Rican and Mexican Latinx adults living in the continental US as ‘immigrants’ because (1) Puerto Rican adults living in the continental US are all US citizens at birth regardless whether they are born in the island or mainland US, and (2) Mexican adults living in the continental US may include a combination of US-born and immigrant populations. In general, the population of focus is unclear in the Abstract.

**RESPONSE: Thank you for this comment, we agree. We have updated the Abstract to reflect these suggestions (e.g., we revised the first sentence by replacing “Latinx” with “Puerto Ricans and Mexican immigrants”). We have also clarified our terminology regarding our sample (e.g., Puerto Ricans and Mexicans born outside (but living in) the continental U.S.) and we restrict our use of Latinx to discussions of the larger Latinx population.**

6. Introduction: 1st paragraph, Page 3: It is unclear how prevalence of ACEs among Latinxs compare to other groups to assess whether 77% is more or less than average. It is also unclear what Latinx groups that this 77% includes – gender/sexual minorities, Mexican/Puerto Rican only as in the study, immigrant Latinx (non-US born), US-born only, etc. Do these adversities occur among US-born?

**RESPONSE: Thank you. We have revised the second sentence on p. 3 as follows, “*Past research has indicated that Latinx individuals are disproportionately affected by certain types of lifetime adversities as compared to non-Latinx whites [4]. A recent study, consisting of foreign-born and U.S.-born Latinxs living in the U.S., found that 77% of participants reported experiencing lifetime adversity during childhood (i.e., adverse childhood experiences; ACEs), and nearly 30% of these individuals experienced four or more adverse events [2].”***

7. Does perceived discrimination refer to interpersonal or structural as this construct has evolved considerably to tease apart different forms of discrimination and their impacts on health. Clearer definitions and statements should be provided according to the specific groups, terminology, and literature referenced.

**RESPONSE: This is an important suggestion. We have added content on p. 6 to address your comment, “*Perceived discrimination refers to unfair treatment based on one’s ethnic/racial background that is rooted in beliefs about the racial superiority of certain groups (e.g., white individuals; 25]. Discrimination can occur in a range of environments, such as at an interpersonal level (e.g., discriminatory comments) or a structural level (e.g., discriminatory policies). For the purposes of the present study, we focused on interpersonal discrimination that occurs over the course of one’s life, which we refer to as perceived discrimination.”***

8. 2nd paragraph, Page 3: It seems the LCT is insufficient to be the sole theory guiding this study. LCT guides the expected impacts of childhood exposures on outcomes in adulthood, including identification of sensitive periods, but doesn’t capture life course experiences/adversity that are specific to Latinx populations, that say critical race theory or weathering hypotheses could. Research by Maggie Alegria, William Vega, Hector Bird, etc. that have examined mental health epidemiology among Latinx populations specifically across the life course may support this background.

**RESPONSE: Thank you for this suggestion. We have incorporated Latino critical theory to supplement life course theory in addressing the importance of race/ethnicity on p. 4, “*Latino critical theory also informed our study hypotheses [8]. Latino critical theory is an extension of critical race theory and examines the ways in which racism, sexism, classism, and other forms of oppression interact to impact people’s lives. This theory (along with past research) informed our decision to include cultural stressors (e.g., discrimination) in our assessment of lifetime adversity. Moreover, based on the numerous differences that exist between Puerto Ricans and Mexican immigrants (e.g., immigration status, prevalence of discrimination), we assessed these groups separately to examine potential differences in exposure and response to lifetime adversity*.”**

9. 3rd paragraph, Page 4-5: What is missing from childhood adversity is the validity of these measures to non-White groups thereby the focus of past research among non-white samples only. To what extent are Latinx sample included in ACE measurement studies?

**RESPONSE: Thank you for your comment, this is an important concern. We have added the following content on p. 5-6, “*Second, the original ACEs scale did not include several types of adversity relevant to Latinxs (or other ethnically/racially minoritized groups), such as discrimination.”***

10. Also, there is more recent theory about dimensionality of ACE’s effects on the brain, versus cumulative effects, that would be important to include in the background of this current study. See work by Katie A. McClaughlin et al., particularly PMID: 27773969. Finally, concerning adult adversity, literature about epigenetic effects could be included here as well as literature examining trauma which is much more developed and has demonstrated similar interactive effects between childhood ACE’s and traumas/adversities during adulthood and their effects on mental health (e.g., disaster-related and veterans-related research examining ACE’s plus adulthood adversities and their impacts on risk for post- traumatic stress).

**RESPONSE: Thank you for this important suggestion. We have incorporated the suggested reference on p. 4-5, “*Third, subgroups of individuals often experienced specific patterns of adversity and these exposure patterns influenced their risk for mental health problems (e.g., dimensional and cluster-based approaches to adversity) [14-17].”* Additionally, we added content regarding the co-occurring nature of adversity across the lifetime, such as in veteran samples: “*Moreover, prior research with military populations has found higher prevalence of ACEs among veterans as compared to civilians [e.g., 15].”***

11. 5th paragraph, page 5-6: The background on discrimination would benefit from describing the different kinds of discrimination (interpersonal/individual versus structural) and their impacts on health. Then it should be explicitly clear that this study examines individual only. It is also noted variation in experiences based on race/skin color within Latinx groups. To readers unfamiliar, it may be important to note the diversity within the Latinx group by race, ethnicity, language use, immigration status, geography, etc. that inform discrimination experiences. Also wouldn’t we expect intersectionality to matter here for Latinx who are black, immigrant, and/or gender/sexual minority such that interacting, multi-layered, co- occurring discrimination experiences present unique threats to health versus discrimination among white-passing, cis-gendered, US-born Latinx populations? Greater acknowledgement of these intersections can help move the knowledge base forward in this area (see original work by Kimberle Crenshaw for more information).

**RESPONSE: Thank you. We added content about discrimination and clarified our focus on interpersonal discrimination on p. 6, “*Perceived discrimination refers to unfair treatment based on one’s ethnic/racial background that is rooted in beliefs about the racial superiority of certain groups (e.g., white individuals; 25]. Discrimination can occur in a range of environments, such as at an interpersonal level (e.g., discriminatory comments) or a structural level (e.g., discriminatory policies). For the purposes of the present study, we focused on interpersonal discrimination that occurs over the course of one’s life, which we refer to as perceived discrimination*.”**

**To be more explicit about diversity between and within Latinx groups, we added content in the first paragraph of p. 8, “*The present study aimed to build on Authors’ [36] study using a person-centered approach to determine whether unique combinations of lifetime adversity were associated with distinct mental health outcomes for Latinxs born outside the continental U.S. Specifically, our goal was to conduct a detailed comparison of two Latinx groups to assess whether differences in exposure patterns to lifetime adversity influenced individuals’ responses to adversities within these two groups. Therefore, we selected Puerto Ricans and Mexicans born outside the continental U.S. because they represented some of variability that exists between U.S. Latinx groups (e.g., immigrant status, skin color, acculturation levels) and are the two largest Latinx ethnic groups living in the U.S. Although these groups share a common language, there are many within-group differences that may influence their perception and experience of adversity [37]. Few studies have conducted direct comparisons between Puerto Ricans and Mexicans born outside the continental U.S., nonetheless, past research comparing Latinx groups more broadly suggests that differences may exist.”* We go on to discuss in more detail the within and between-group differences among Latinx groups.**

**To highlight the intersectional nature of individual characteristics with Latinx populations, we added two sentences in the last paragraph of p. 9, “*In addition to isolating the effects of individual characteristics alone, it is important to understand the overlapping influences of these types of characteristics on health outcomes. One useful approach to identifying the ways in which different characteristics interact to influence response to risk is by using latent profile analysis*.”**

12. Concerning Latinx heterogeneity, this text should be introduced much earlier to help guide the reader as to why we would expect variation in the application of ACEs and LCT in research pertaining to Latinx populations.

**RESPONSE: Thank you for this comment. We have added the following content on p. 4, “*Moreover, based on the numerous differences that exist between Puerto Ricans and Mexican immigrants (e.g., immigration status, prevalence of discrimination), we assessed these groups separately to examine potential differences in exposure and response to lifetime adversity.”***

13. Also, it is unclear why Mexican and Puerto Rican groups are of focus – is only because they are the largest sub-groups or is it that other groups are underrepresented in research? How does research on Mexican and Puerto Rican populations generalize to other groups? What are the public mental health impacts of focusing on the two largest sub-groups only? How do these two groups differ in terms of other socio-demographics such as race, age, immigration status, language use, etc. (PEW Hispanic Research Center may provide this information). Why determine highest risk groups for tailored intervention versus low-dose, ubiquitous interventions for all (e.g., universal mental health coverage, Spanish-language competent providers, etc.) – what are the tradeoffs of one approach versus another? These motivations should be made clearer.

**RESPONSE: These are important comments, thank you. We have added content to clarify the justification for our sample. See our response to comment #11.**

**We agree that it is important to identify those at highest and lowest risk to inform targeted intervention approaches. We clarified this on p. 9, “*This information could be used to help determine subgroups at highest and lowest risk for mental health problems and inform preventive interventions tailored to the individuals’ pattern of risk exposure*,” as well as in several other places in the manuscript.**

**We added the following content to discuss the universal vs. tailored approaches on p. 30, “*Results from our study also suggest that different patterns of adversity exposure are linked to different levels of mental health outcomes, and therefore, may require different intervention dosage. For example, Mexicans experiencing abuse, neglect and discrimination or high ACEs and high discrimination may need a distinct and more intensive intervention than Mexicans exposed to high ACEs alone. Using a universal approach to prevention, in which everyone gets the same intervention, may be less effective in such cases where the needs of individuals may vary. Understanding which individuals are at the highest and lowest risk for mental health problems is critical for developing tailored programs to intervene across these risk levels.”***

14. Methods: Sample, Page 9: It is unclear the motivation for restricting the sample to two Latinx groups only. It is also unclear from the Abstract and Introduction if this study and Background is pertaining to Puerto Rican and Mexican adults living in the mainland US who were not born in the mainland US, specifically. The only reason provided, “These were two of the largest Latinx subgroups in the sample and represent the two largest Latinx subgroups living in the U.S.” is still unclear in terms of what is meant by “subgroup”. Ethnic subgroup? Among immigrant populations? Among non-mainland US born populations? These definitions and rationale for inclusion criterion are unclear yet they should be explicit throughout the manuscript.

**RESPONSE: Thank you. See our response to the previous comment #11. Additionally, we revised to manuscript to use greater precision and consistency in our terminology describing our sample (e.g., “Puerto Rican and Mexicans born outside the continental U.S.”).**

15. Also, if 1753 of the 4645 were included only, how did the excluded sample compare to those that were included? Why were they excluded and why not assess South American and Central American and Caribbean groups separately?

**RESPONSE: Please refer to our response to comment #11 for a justification for our sample. We also felt that treating Latinxs from South America or Central America as two large groups overlooks the immense variability that exist within these populations.**

16. Page 9-10: What is the validity of the ACE, discrimination, and mental health measures among the included Latinx populations in this study, specifically? That is, Puerto Rican and Mexican adults living in the mainland US who were not born in the mainland US. Also, alpha reliability of each measure would be helpful to include in the text.

**RESPONSE: Thank you for this comment. These measures have been used with U.S. Latinx populations broadly (i.e., majority Mexican samples), but few have assessed the validity within exclusively Mexican or exclusively Puerto Rican samples. We have included the following text in the limitations section on p. 32, “*Sixth, although our measures had been validated with Latinx populations, few had been validated with our exact sample (i.e., Puerto Ricans and Mexicans born outside, but living in, the continental U.S.). However, this is a limitation to the broader field of mental health research, as more studies are needed to determine the validity of mental health measures across various Latinx populations*.”**

**It seems somewhat repetitive to have the reliabilities of each scale for Puerto Ricans and Mexicans listed in the text as well as in Table form. In our opinion, the Table presents a clear overview of the internal consistencies as well as other descriptive statistics.**

17. Are there covariates available to assess racial/skin color variation as this would be highly relevant to the discrimination measures? Also, potential buffers or resilience factors such as health insurance status, education, and close relationships that mitigate risks of adversity/discrimination on mental health should be included. Further, is state/geography examined as CA/NY present more immigrant-favorable policies versus FL, etc. (e.g., Hatzenbuehler et al. study cited regarding state immigration policies and mental health).

**RESPONSE: The one race item in the dataset was not a useful covariate for the present sample, as 50% of Mexican immigrants did not respond (i.e., either refused or selected the unknown/no response option) and about 40% of Puerto Ricans did not respond. This indicates that the survey items did not adequately capture many participants’ conceptualizations of their racial identity, and therefore, we were unable to reliably assess the impact of racial identity or skin tone.**

**Based on your suggestions, we added health insurance status, education level, and social support as covariates and updated our analyses.**

**We have revised our limitations section to clarify the samples to which our study findings can be generalized. For example, on p. 30, we stated, “*First, there were numerous demographic constraints to the study. For example, the sample we used from the HCHS/SOL SCAS data was comprised of Puerto Ricans and Mexicans born outside the continental U.S. living in well-established urban immigrant destinations (i.e., Chicago, Miami, San Diego, the Bronx), primarily middle-aged adults that have lived in the continental U.S. for an average of over 20 years. Therefore, our findings may not generalize to other Latinx populations outside of our sample (e.g., younger adults, recent migrants, individuals living in other areas of the U.S., continental U.S.-born individuals, Latinxs born in other parts of Latin America).*”**

18. Discussion: Page 25-26: US citizenship status comes with greater access to health insurance as well, therefore, access to mental health care, which can mitigate negative effects of discrimination on mental health among Puerto Ricans. In contrast, those without access or without a public insurance option may be more prevalent among Mexican immigrants, particularly those that are undocumented. Nevertheless, racial/skin color variation isn’t taken into account here when describing differences between Puerto Rican and Mexican discrimination experiences.

**RESPONSE: These are great comments, thank you. We have revised the end of the first paragraph on p. 29 as follows, “Additionally, Puerto Ricans in our study had higher levels of health insurance coverage. Therefore, despite experiencing relatively high levels of discrimination, the impact on their mental health might be minimized given that being a U.S. citizen protects them from the fear of being deported or not hired due to their documentation status and allows for access to health insurance.”**

**In regards to skin color variation, we have included content in our limitations section: “*Fifth, we were unable to assess the effects of skin color variation or racial identity on the relation between discrimination and mental health outcomes, which past literature indicates to be an important factor to consider [8].”***

19. Limitations of the sample should be elaborated as this is a highly specific sample. There are many unique Latinx groups excluded from this study including, but not limited to, US-born Latinx, Gen Z and Millennial Latinx, gender/sexual minorities (gender assessed as binary variable), no racial variation captured, rural/suburban Latinx populations (different from new settlement areas as indicated), indigenous Latinx, as well as Central/South American and Other Caribbean groups. That US-born, young Latinx are excluded limits generalizability of these findings to understanding ACE’s that occur in the continental US context, specifically. The ACE’s captured in this study reflect those that occur in Mexico or the island of Puerto Rico, and *not* childhood adversities that are experienced in the continental US for other Latinx populations. Further, time spent back and forth between Mexico or Puerto Rico and mainland US during childhood, especially for Puerto Ricans who are citizens and arguably have easier migration abilities, but also Mexican border populations, isn’t factored into this study. These experiences could introduce a unique set of migration-related ACE’s that are not captured by the ACE measure. These important limitations should be noted. These exclusions to the sample should also be noted, and as such, use of Latinx should be re-considered or defined.

**RESPONSE: Thank you, we agree. We have revised the limitation section to reflect these considerations. See our response to comments 17-18.**

**As described above, we have clarified our terminology regarding our sample (e.g., Puerto Ricans and Mexicans born outside (but living in) the continental U.S.) and we restrict our use of Latinx to discussions of the larger Latinx population.**
