# Supplementary material for: Puerto Ricans and Mexican immigrants differ in their psychological responses to patterns of lifetime adversity
Source: PLoS One. 2021 Oct 18;16(10):e0258324. doi: 10.1371/journal.pone.0258324 (PMC8523059; doi:10.1371/journal.pone.0258324)
Supplement: Attachment — Submitted filename: Response to Reviewers.docx [file pone.0258324.s003.docx]

**Reviewers' comments**

**Reviewer #1**

1. Table 6: While the text says that "As compared to those who preferred speaking Spanish, those who preferred speaking English had greater odds of belonging to the Dual Exposure and Maltreatment and Discrimination profiles as compared to the Low Exposure profile." the respective line in Table 6 is named "Prefer Spanish". That is confusing and I would suggest to change the name of the line in "Prefer English". Moreover, I wondered whether the categorization of Spanish=0 and English =1 is appropriate for the analyses of Odds ratios, since -to the extent I know- no meaningful Odds ratio can be determined for a category 0. Please consider changing the categorizations using the values 1 and 2.

**RESPONSE: We appreciate this comment. We have revised the variable in Table 6 to “Prefer English,” thank you for catching this. Regarding the second aspect of this comment, our understanding is that the values assigned to each group (e.g., “0” and “1” vs. “1” and “2”) does not matter, they are merely to distinguish the two groups from one another for the purposes of analysis and would not change the significance or effect sizes of the results. We are happy to provide more background on this if the reviewer requests it.**

2. Results on the associations between latent risk profiles and mental health in Puerto Ricans: The non-significance of the (possible) differences for the Dual Exposure group may be a result of the small sample size of this sub-group (with n=35?) and the lack of statistical power. Please discuss this possibility.

**RESPONSE: Thank you for this comment. We agree that the Dual Exposure profile’s smaller sample size limited power to compare across profiles. However, the findings we highlighted in the discussion section focused on the fact that the ACEs Only Profile had similar (and actually higher levels of mental health problems, although not reaching significance) levels of mental health problems as compared to the Dual Exposure Profile. This was contrary to our hypothesis that experiencing a combination of discrimination and childhood adversity would be associated with the greatest mental health problems. We believe that adding content describing the non-significant differences we found between the Dual Exposure and Low Exposure profiles (for Puerto Ricans) in the discussion section would be less interesting and relevant for readers.**

**Reviewer #3**

3. The introduction needs to have a more straightforward argument because there is not enough emphasis on this paper's contribution from the start.

**RESPONSE: Thank you for this comment. We have added content on the first page to make the goal and significance of this paper clearer from the outset, “*This information is critical for improving the understanding of these co-occurring determinants of health and for developing interventions to interrupt their effects. For example, identifying subgroups at lowest and highest risk for various mental health problems can help inform decisions about who is at greatest need and who could benefit most from intervention. Analyzing Puerto Ricans and Mexicans separately allows for further nuance to our understanding of risk processes.*”**

4. Additionally, the childhood adversity section should explicitly state why Latinx people are impacted differently in adulthood and why this outcome matters. The paper remains vague in this section of the literature.

**RESPONSE: We appreciate this comment. To clarify, our argument is not that the long-term effects of childhood adversity are different for Latinx vs. non-Latinx groups, rather, that exposure to childhood adversity in combination with adult adversity is important to assess in this population because of the high prevalence of various lifetime adversities. We added content to clarify this on p. 6, “*This could be particularly important for Latinx populations, who often experience numerous stressors throughout their lifetimes (e.g., perceived discrimination, economic stress). Studying child adversity in isolation of adult adversity limits our ability to identify the collective influence of adversity across the lifespan*.”**

5. The perceived discrimination section lacks the critical race framework that exists in the literature regarding the racialization of Mexican and Puerto Ricans. These perceived discriminatory practices also referred to as racial microaggressions, are rooted in the anti-blackness in the United States. It is also essential to discuss the colorism that plays a role in these micro-aggressions. For some Latinx people with lighter complexions, discrimination does not occur until they leave their home country.

**RESPONSE: Thank you. We added content in this section to highlight the role of anti-black racism and colorism in Latinxs’ experiences of perceived discrimination: “For Latinxs born outside (but living in) the continental U.S., perceived discrimination can be related to anti-immigrant and/or anti-black beliefs. In 2019, nearly 60% of U.S. Latinxs reported experiencing perceived discrimination at least “from time to time,” and even higher rates were reported for those with darker skin, highlighting the role of colorism and anti-blackness in these acts of discrimination [5].”**

6. Further, the result and implications need to discuss the racialization of Latinx people because these sections need to connect the critical race frameworks that exist in Latinx studies.

**RESPONSE: We appreciate the reviewer’s continued attention to contextualizing the study findings within critical perspectives, and we agree this could be more explicit. In addition to the content we added in the previous revision (e.g., “*In fact, Latino critical scholars argued that individuals’ experiences of the world are shaped by various intersecting aspects of their identity, such as citizenship status [8]*.”), we added additional content in the discussion and implications section to address this feedback. For example, in the second to last paragraph in the discussion section we added, “*These findings underscore the importance of adopting a critical, intersectional approach to understanding within- and between-group differences among Latinxs*.” In the implications section, we added, “*Understanding the effects of discrimination is particularly important in light of past research documenting the racialization of Latinx immigrants, a process that often associates Latinx immigrants with criminality [72].*” Please let us know if you would like to see additional content included.**
